# The etiology and clinical features of non-CAH primary adrenal insufficiency in children

**DOI:** 10.3389/fped.2022.961268

**Published:** 2022-08-19

**Authors:** Ziqin Liu, Yi Liu, Kang Gao, Xiaobo Chen

**Affiliations:** Department of Endocrinology, Children's Hospital Capital Institute of Pediatrics, Beijing, China

**Keywords:** adrenal, primary adrenal insufficiency, mutation, children, etiology

## Abstract

**Background:**

The most common cause of primary adrenal insufficiency (PAI) in children is congenital adrenal hyperplasia; however, other genetic causes occur. There is limited epidemiological and clinical information regarding non-CAH PAI.

**Methods:**

Data for patients diagnosed from January 2015 to December 2021 at a tertiary hospital in northern China were retrospectively analyzed. We excluded those with CAH, which is the most common pathogenic disease among PAI patients. Next-generation sequencing was used for genetic analysis.

**Results:**

This retrospective study included 16 children (14 males and 2 females) with PAI. A genetic diagnosis was obtained for 14/16 (87.5%) individuals. Pathogenic variants occurred in 6 genes, including ABCD1 (6/16, 37.5%), NR0B1 (4/16, 25.0%), NR5A1/steroidogenic factor-1 (2/16; 12.5%), AAAS (1/16, 6.25%), and NNT (1/16, 6.25%). No genetic cause of PAI diagnosis was found in 2 girls (2/16, 12.5%).

**Conclusions:**

Causes of PAI in children are diverse and predominantly affect males. Most PAI in children is congenital, and ABCD1 gene defects account for the largest proportion of PAI cases. Whole-exome sequencing is a tool for diagnosis. However, diagnoses are unclear in some cases.

## Introduction

Primary adrenal insufficiency (PAI) is classified as a disease intrinsic to the adrenal cortex. The clinical manifestations of PAI result from a deficiency in one to three adrenocortical hormones (aldosterone, cortisol and androgens) (1). In adults, most cases of PAI are caused by autoimmune adrenalitis; however, in children, genetic causes of PAI play more important roles (2), especially in neonates and infants (3). The etiologic spectrum of PAI is broad and includes congenital adrenal hyperplasia (CAH), X-linked Adrenal hypoplasia congenita, SF-1 linked Adrenal hypoplasia congenita, adrenoleukodystrophy (ALD), adrenomyeloneuropathy, familial glucocorticoid deficiency (FGD), Triple A syndrome, Xp21 deletion syndrome, infectious adrenalitis and autoimmune adrenalitis (4–7). With the exception of CAH, each etiology is rare, and there are few studies on their clinical and laboratory features. PAI ranges from mild non-specific symptoms to life-threatening shock, and clinicians must maintain a high level of suspicion for this disease (8). The diagnostic investigation process, although well established, can be challenging. The epidemiology of PAI in children is not well defined. The present study aimed to summarize the etiological spectrum, clinical manifestations, laboratory features and management of non-CAH patients followed in the pediatric endocrinology department of a tertiary hospital over 7 years.

## Materials and methods

### Patients

**Figure 1:**
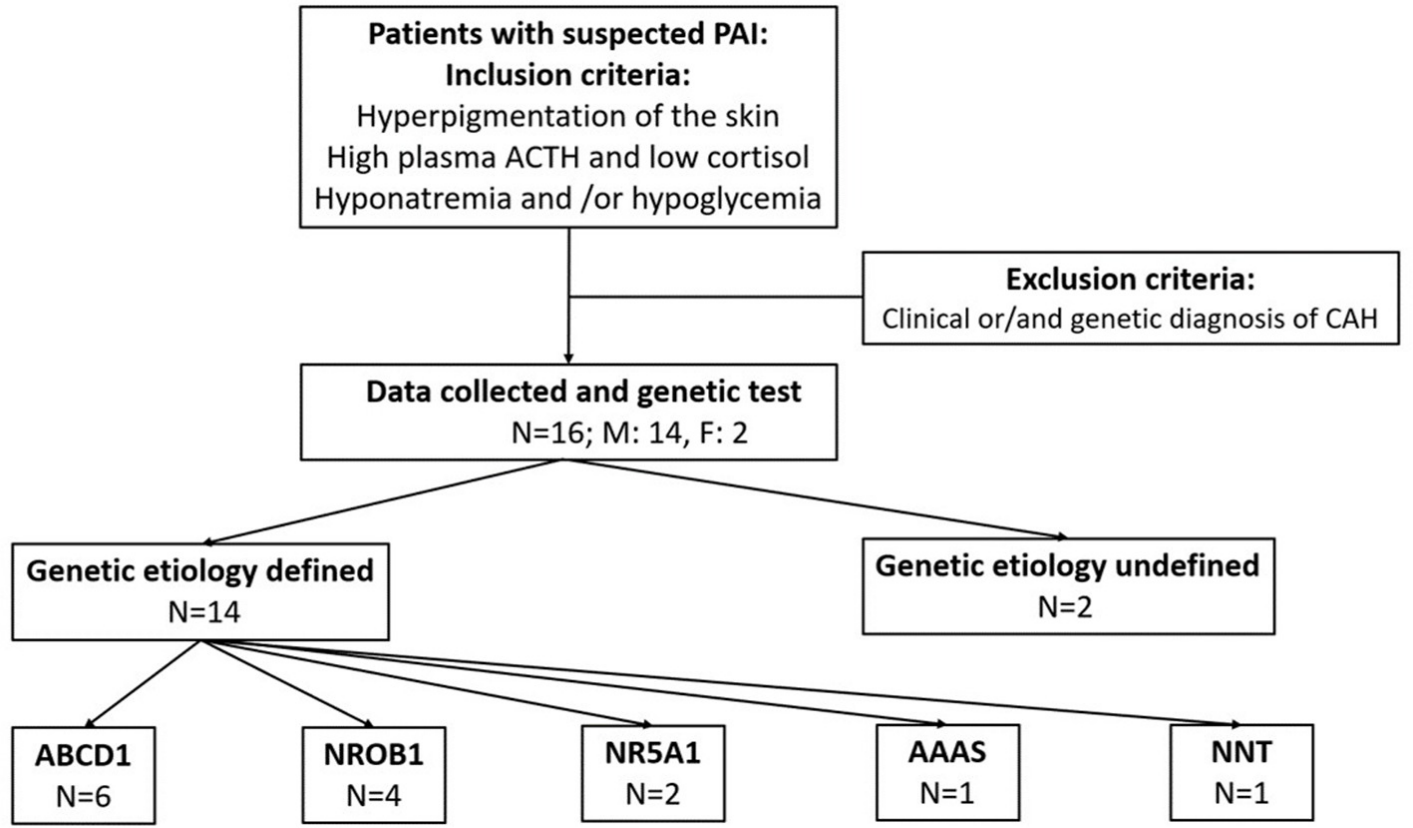
Study design, recruitment and genetic results.

This was a retrospective study of PAI conducted from January 2015 to December 2021 at Capital Institute of Pediatrics (CIP). The patients were outpatients at CIP and other referral centers (general hospitals in Beijing and some general hospitals in northern China when PAI was suspected). The inclusion criteria were coexistence of the following criteria: (1) clinical symptoms/signs suggestive of PAI (recurrent hypoglycemia, hyperpigmentation of skin, hyponatremia with hyperkalemia); (2) a low serum cortisol concentration (<3 μg/dl) in a blood sample taken in the early morning strongly suggestive of adrenal insufficiency (9, 10); and (3) 8 am plasma corticotropin concentration (ACTH) higher than 2 times the normal upper limit (normal range: 6–63 pg/ml). The exclusion criteria were as follows: (1) congenital adrenal hyperplasia (CAH) and (2) incomplete clinical data (Figure 1). Informed consent was obtained from all individuals included in this study. The study protocol was approved by the Children's Hospital Capital Institute of Pediatrics Ethics Committee (SHERLL2018005-01, date: 13.03.2018–12.03.2022).

### Genetic analysis

DNA samples obtained from the family were sequenced to identify the causal gene using whole-exome sequencing (WES). DNA was isolated from peripheral blood using a DNA Isolation Kit (Bioteke Corporation, AU1802, Wuxi, China). Genomic DNA samples (1 μg) were fragmented into 200–300 bp fragments using a Covaris Acoustic System (Covaris, Woburn, MA, USA). The DNA fragments were then processed by end repair, A-tailing, adaptor ligation and four-cycle precapture polymerase chain reaction amplification, after which all exons and the 50 bp bases in their adjacent introns were captured by a SeqCap EZ Med Exome Enrichment Kit (Roche, Madison, WI, USA). Postcapture amplification and purification were performed on the DNA library, and then sequencing was performed on an Illumina HiSeq X Ten platform (Illumina, San Diego, CA, USA). The raw data produced were then filtered and aligned with the human genome reference (hg19) using the BWA Aligner (http://bio-bwa.sourceforge.net/). Variants were identified using NextGene V2.3.4 software (Soft genetics, LLC, State College PA, USA). The data had a 151.24 × mean read depth, and ~97.95% of the target bases were covered at a 20× average read depth. The filtered variants were then annotated by using NextGene V2.3.4 and the laboratory's own scripts to obtain related information, including the conservation of nucleotide bases and amino acids, prediction of the biological functions, frequency in normal populations (compared with 1,000 Genomes, ExAC, dbSNP database and local specific databases), and the data from HGMD, ClinVar and OMIM databases. The pathogenicity of all variants was interpreted and categorized according to ACMG (11) standards.

## Results

### Clinical characteristics

**Figure 2:**
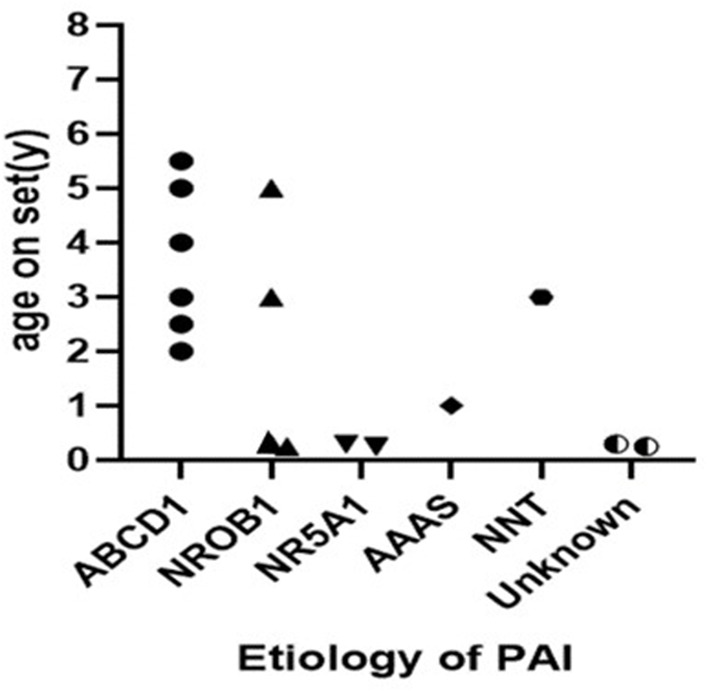
The age onset of patients with PAI.

The study included 16 patients (14 males and 2 females) with a median age of 2.3 (p25:0.3, p75:4.8) years at the time of onset (Figure 2). Males significantly outnumbered females by a ratio of 7 to 1. All 16 cases involved pigmentation. Three individuals had epilepsy, 3 individuals had a micropenis, and 1 had cryptorchidism. Two individuals had recurrent infections and asthenia. Vision loss, short stature, ventricular septal defect and grade I AVB were also observed (Tables 1, 2).

**Table 1:** Clinical manifestations of patients with PAI.

**No**	**Sex**	**Age onset (y)**	**BW g**	**BH cm**	**Clinical presentations besides PAI**	**H mg/m^2^**	**Flu mg**	**Other treatment**	**Following up**
1	M	3.0	2,400		Unilateral cryptorchidism, adrenomyeloneuropathy	11.7			
2	M	5.0			Adrenomyeloneuropathy	20.0		Sodium valproate	Died
3	M	2.5			Adrenomyeloneuropathy, asthenia	15.0		Bone marrow transplant	
4	M	5.5			Diminution of vision, adrenomyeloneuropathy	12.0			
5	M	4.0	3,700		Vision loss, sensineural deafness, cerebral ALD	8.8			
6	M	2.0	2,750		Adrenomyeloneuropathy	12.7		Bone marrow transplant	
7	M	5.0	2,850	50	Recurrent infection, asthenia	12.5			
8	M	1.0	2,900		Achalasia, micropenis, short stature	15.0			
9	M	5.0	3,600	50	Recurrent respiratory tract infection	12.5			
10	M	0.1	4,580	55	Ventricular septal defect	16.6	0.1		
11	M	3.0	3,550		Micropenis	15.0	0.1		
12	M	0.25	4,330		First block atrioventricular block	31.0	0.2		
13	M	0.01	2,470	49	Micropenis	26.0	0.2		
14	M	0.25	3,300	50	Micropenis	30.0	0.2		
15	F	0.16	2,700	45	Epilepsy, mental retardation, short stature	10.0	0.1	Sodium valproate	
16	F	0.3	2,300	46	Epilepsy, mental retardation, short stature	11.8	0.2	Phenobarbital	

BW, birth weight; BH, birth height; H, hydrocortisone; Flu, fluhydrocortisone.

**Table 2:** Lab tests and genetic tests of patients with PAI.

**No**	**Na** **mmol/l**	**K** **mmol/l**	**Glucose** **mmol/l**	**ACTH** **pg/ml**	**Cortisol** **ug/dl**	**Gene**	**DNA mutation**	**Protein change**
1	139	4.4	5.1	2,000	2.36	ABCD1	c.1534G>A	p.G512S
2	134	3.5	5.2	2,000	0.37	ABCD1	**c.1376_1377insC**	p.Leu461Profs*95
3	140	4.0	4.9	1,000	2.40	ABCD1	**c.1376_1377insC**	p.Leu461Profs*95
4	143	4.1	4.9	974	4.99	ABCD1	c.1544C>T	p.S515F
5	140	3.9	4.8	256	6.18	ABCD1	**c.445A>T**	**p.S149C**
6	142	4.2	4.9	1,154	0.45	ABCD1	**c.626C>A**	**p.A209D**
7	130	4.5	4.7	2,000	1.64	NROB1	**c.793A>C**	**p.T265P**
8	140	4.8	4.4	2,000	1.20	AAAS	c.580C>T, c.580C>T	p.R194*, p.R194*
9	139	4.1	4.3	2,000	0.05	NNT	**c.2902C>T, c.1745T>C**	p.R968*, p.L582P
10	128	6.2	5.2	312	3.98	NROB1	c.884T>G	p.L295R
11	128	4.0	4.7	2,000	1.33	NROB1	c.704G>A	p.W235*
12	120	7.8	2.9	2,000	1.27	NROB1	**c.609delT**	p.E204Kfs*60
13	118	7.9	2.8	1,250	1.20	NR5A1	**c.1104C>A**	P.C368*
14	104	6.4	3.7	570	2.10	NR5A1	c.104G>A	p.G35D
15	124	3.7	1.8	2,000	0.84	Unknown		
16	104	6.4	6.3	637	2.41	Unknown		

A molecular genetic diagnosis was obtained for 87.5% (14/16) of the children with PAI. A molecular diagnosis was obtained for all of the male patients, whereas no pathogenic mutation was found in the 2 female patients. Six different gene mutations were found in this cohort. ABCD1 accounted for 37.5% (6/16) of PAI, NR0B1 for 25.0% (4/16), NR5A1/steroidogenic factor-1 for 12.5% (2/16), and AAAS and NNT for 6.25% each (1/16). Each genetic diagnosis is shown in Table 2.

### Adrenoleukodystrophy

ALD was the most common etiology, constituting 37.5% (6/16) of all cases and 42.9% (6/14) of all cases among the boys. Six of the patients were diagnosed with ALD and classified as childhood cerebral ALD (CCALD). Two of them were brothers, and the elder brother died due to encephalopathy. The median age at onset of PAI was 3.7 (2.0–5.5) years among ALD patients. All of the patients presented with neurological complications (Table 3); vision loss, sensineural deafness and cryptorchidism were also present in some cases. None of the adrenoleukodystrophy patients showed salt wasting. Plasma very-long change fatty acid (VLCFA) level analyses revealed high plasma C26 levels. Five heterozygous mutations in the ABCD1 gene were found. Patients 2 and 3 were siblings (Figure 2).

**Table 3:** Composition of primary diseases in different countries or regions.

**Country/City**	**UK (12)**	**Turkey (13)**	**Japan (14)**	**Turkey (15)**	**Italy (16)**	**China/Guang Zhou (17)**	**China/Shang Hai (18)**	**China/Bei Jing**
Time of duration	25 years		15 years	21 years	20 years	29 years	5 years	7 years
Number of patients	150	84	44	22	111	49	11	16
Number/year	6		2.93	1.04	5.55	1.68	2.20	2.28
Non-CAH ratio				30.1%	13.8%	11.3%	15.5%	10.6%
Male to female					77:34	47:2	9:2	7:1
ABCD1	Excluded	2/excluded typical	Excluded	8	25	22	1	6
NROB1	12	12/excluded	18	1	16	20	2	4
SF-1	1	1	0			1	0	2
MC2R	30	25	1	3	4	0	0	0
MRAP	7	9	0		1	0	0	0
AAAS	11	1/excluded typical	2	5	7	2	3	1
NNT	10	7	2	0	0	0	1	1
AIRE	Excluded	Excluded	0	1	25	3	0	0
SAMD9	5	0	7	0	0	0	0	0
CDKN1C	2	0	1	0	0	0	0	0
CYP11A1	12	9	0	0	0	0	0	0
TXNRD2	7	0	0	0	0	0	0	0
Acquired PAI	0	0	0	0	3	1	0	0
Pearson syndrome	0	0	0	0	1	0	0	0
Autoimmune PAI	0	0	0	0	20	0	0	0
Unknown	53	18	13	3+1?	9	0	3	2

### Congenital adrenal hypoplasia

The analysis showed that 4 boys had NR0B1 gene mutations. The age of onset ranged from birth to 5 years old. Patient 7 had an older brother who experienced unexplained septic shock and died at 19 years of age. A retrospective analysis of his case data showed that adrenal crisis was a more reasonable diagnosis. Three patients experienced a salt-wasting crisis shortly after birth and profound hypoglycemia in infancy. ACTH levels were between 312 and 2,000 pg/ml and cortisol levels between 1.24 and 2.98 μg/ml at diagnosis. Blood sodium of the children was between 120 and 130 mmol/l, and blood glucose was between 2.8 and 4.7 mmol/l. Two boys were found to have a pathogenic variant in NR5A1. Both mutations were missense mutations (Figure 2). They showed salt-wasting shortly after birth. Both were found to have obvious male genitalia but with a micropenis. Their karyotype was 46, XY.

### NNT and AAAS

We found 1 boy with NNT mutations and 1 boy with an AAAS mutation. Both mutations were classified as causative of familial glucocorticoid deficiency. An 8-year-old boy presented with a 5-year history of hyperpigmentation. The patient's mother also experienced an early miscarriage. He had a healthy 5-year-old younger brother. This patient's clinical manifestations were consistent with PAI. Two novel NNT mutations were observed in this patient with only glucocorticoid deficiency. The genetic analysis revealed heterozygous mutations c.2902C>T, p. R968^*^ and c.1745T>C, p. L582P in NNT.

An 11-year-old boy was admitted to the hospital with a 10-year history of hyperpigmentation. The patient also experienced excessive dry mouth symptoms. His other signs included short stature, osteoporosis, microcephaly and a micropenis with Tanner stage I. Antibodies against insulin and thyroid were negative. IgA, IgM, and IgG levels were normal. His ACTH level was 2,000 pg/ml, while his cortisol level was 1.2 μg/ml (8 a.m.). The genetic analysis confirmed a pathogenic homozygous mutation in the AAAS gene c.580C>T, p. R194^*^.

### Unknown etiology

Two female patients were diagnosed with non-CAH PAI of unknown etiology. Patient 15 was admitted to the hospital at 2 months of age due to an afebrile seizure and hyperpigmentation. She was found to have extreme hyponatremia and hypoglycemia. Her baseline labs showed a serum sodium level of 124 mmol/l, serum potassium level of 3.7 mmol/l, and serum glucose level of 2.4 mmol/l. Initial investigations showed a low cortisol level of 0.05 μg/ml with an ACTH level 2,000 pg/ml. Patient 16 was first admitted to a hospital because of feeding difficulty. She was 3 months old at that time with hyperpigmentation. Her cortisol was 2.41 ug/ml with an ACTH level of 637 pg/ml. She was not found to have hypoglycemia or electrolyte disturbance during the inpatient stay. She was administered hydrocortisone 11.8 mg/m^2^. Both female patients' liver function, kidney function and 17-OHP levels were normal. Their chromosomal karyotypes were 46, XX (20). Their adrenal enhanced CT showed thin bilateral adrenal glands.

During the follow-up, patient 15 often experienced convulsions after infections in the form of generalized seizures. She was diagnosed with epilepsy and administered sodium valproate. Patient 16 experienced infrequent convulsions. She was also diagnosed with epilepsy and was administered phenobarbital. Both patients had convulsions after infections, and no abnormalities were found by brain MRI. Due to the early onset of PAI, convulsions of unknown origin and a high suspicion of congenital disease, WES and copy number variation analyses were performed, but no pathogenic mutation was found.

## Discussion

Here, we collected data from children with PAI (CAH excluded) at a single center over a 7-year period, and a genetic diagnosis was obtained for 87.5% of these patients. The proportion of males was significantly higher than that of females. The etiology is complicated. ABCD1 gene mutation was the dominant etiology, accounting for 37.5%, followed by NROB1 gene mutation, accounting for 25%. NR5A1, NNT and AAAS mutations were also found.

PAI is rare in different countries and regions (Table 3), with <10 cases reported yearly (12–18). However, this number does not include undiagnosed patients. The signs of PAI vary depending on the type. Hyperpigmentation was the common presentation in our study. Hyperpigmentation of skin, genitalia, lips, gums, and scars is one of the most remarkable signs of PAI, occurring in 90% of affected children (13). Patients may have various presentations, including hyperpigmentation, hypoglycemia and hyperkalemia (19). In some cases, signs of PAI are subtle and non-classical and can be easily overlooked by doctors and caregivers. The more severe the clinical symptoms are, the earlier the diagnosis can be made. In our study, one of the ALD brothers was diagnosed after the other became unwell and eventually died. In addition, one patient had a brother who died of septic shock, and although the brother was not clinically diagnosed, we concluded that he might also have had PAI. Thus, recognition of PAI requires a high index of suspicion, especially when there is family history, and delay in treatment may result in poor clinical outcomes (20).

Sex differences were observed in a UK multicenter cohort study (12), in which there were more affected boys, consistent with the results of our study. Two other Chinese single-center studies also showed that patients were predominantly male (17, 18). A Turkish multicenter study (13) excluded patients with ABCD1 and NROB1 mutations, and the incidence appeared to be approximately the same among boys and girls.

The etiologic spectrum of PAI is highly heterogeneous (Table 3). The proportion of PAI caused by non-CAH is under 20% in the Chinese population (Table 3) (17, 18). The Turkish study revealed that MC2R gene mutations were the first common etiology in PAI, and showed no sex differences in PAI incidence (13). The population studied had a high degree of consanguinity and marked genetic founder effects (13). An English study subsequently revealed that MC2R mutations (adrenocorticotropin receptor; 30/155, 19.4%) were the most common etiology (12). Both large studies excluded ALD. MC2R had the highest percentage of mutations in that report, but there was no single case in our study, and two other studies from China (17, 18) did not detect MC2R mutations. In the present study, ABCD1 was the most common etiology, accounting for 35% of all patients with PAI; the second most common etiology was NROB1, accounting for 25%, which was rare in former studies. In a Japanese study, NROB1 mutations were identified in 78% of male patients (14), and only a single case with MC2R mutations was reported. These results are similar to ours. In addition to MC2R, the reported incidence of another type of familial glucocorticoid deficiency in China is also lower than that in the UK and Turkey, with more patents with MRAP, AAAS or NNT mutation. In an Italian study, 25 patients had APS-1, and 20 had APS-2 or isolated autoimmune PAI (16), accounting for 40.5% of non-CAH PAI cases. This number is much higher than in other reports. As a potential cause of autoimmune PAI in children, AIRE-induced PAI needs to be considered. Based on data from different countries, we believe that the etiology of PAI is ethnically distinct.

In 1976, it was shown that the principal biochemical disorder in X-ALD was the accumulation of VLCFA (21). ALD is an important diagnosis in boys, and VLCFA analysis should be considered. Fifty percent of ABCD1 mutations lead to a truncated ALDP, whereas many missense mutations result in the formation of an unstable protein (22). The disease incidence is estimated to be 1:17,000, 1:21,000 in males, with a disproportion that may reflect the morbidity related to PAI in males preceding diagnosis of X-ALD (23). In a recent international multicenter study, the authors showed data for patients with ALD, and PAI was present in 71.1% (24). This proportion is similar to that observed in our study. The most common mutation is p.Gln472Argfs^*^83 in exon 5 (25). Our 6 patients also mainly had missense mutations; however, none were common mutations.

NROB1 and NR5A1 have emerged as key regulators of adrenal development and are nuclear receptors (26). X-linked AHC might occur in between 1:140,000 and 1:1,200,000 children (27). The true incidence of NROB1 mutations is not currently known. NROB1 mutations are a relatively frequent cause of PAI in boys (29.4%). A late-onset form of X-linked AHC has been described in several cases (28). In addition to point mutations, there are contiguous deletions of genes in Xp21 with loss of the locus for GK, congenital adrenal hypoplasia (AHC) and/or Duchenne's muscular dystrophy (DMD) (29). NR5A1 mutations causing PAI in males are relatively rare. A more common presentation is gonadal dysfunction in 46, XY individuals. Our results were similar to those of other studies (27). However, there was no sexual reversal in our patients.

We also diagnosed a case of FGD and triple A syndrome. The patient was diagnosed with PAI at much older age than others in our study. Alacrima is usually the earliest and most common feature, followed by achalasia. However, both features are easy to miss. Careful analysis of the patient's history and the presence of PAI led to clinical suspicion of triple A syndrome. Most triple A syndrome patients present with achalasia or PAI in the first two decades (30). This presentation distinguishes the disorder from others caused by genetic mutations that lead to PAI in the first two decades of life (30), but autoimmune diseases need to be identified at this stage. Amyotrophy is found in nearly half of affected patients, and long-term follow-up is recommended. In addition, we presented the case of an FGD patient with NNT mutations. There are many clinical issues that need to be addressed in his case. He visited doctors at a local hospital several times, but PAI other than immunodeficiency was not considered.

Obtaining an accurate diagnosis is essential for management; whole-genome sequencing (WGS) will become increasingly available (31), and new genetic causes may emerge as our understanding of human adrenal development expands (32). Genetic counseling should be offered to the family as soon as the genetic diagnosis made. If the mother is confirmed to be a carrier for an ABCD1 mutation, testing should also include all male siblings of the index case (33).

## Conclusion

The genetic causes of PAI in children are diverse. The etiology varies by race. In the non-CAH group in our cohort, ABCD1, NR0B1, NR5A1, AAS, and NNT gene mutations were identified. Despite genetic testing, the cause of postnatal-onset severe PAI is not identified in some patients.

## Data availability statement

The datasets presented in this article are not readily available due to concerns regarding participant/patient anonymity. Requests to access the datasets should be directed to ZL (1934855597@qq.com).

## Ethics statement

The studies involving human participants were reviewed and approved by Capital Institute of Pediatrics Ethics Committee. Written informed consent to participate in this study was provided by the participants' legal guardian/next of kin.

## Author contributions

ZL collected and interpreted the data and wrote the paper. YL and KG collected the clinical data. XC supervised the study. All authors contributed to the article and approved the submitted version.

## Conflict of interest

The authors declare that the research was conducted in the absence of any commercial or financial relationships that could be construed as a potential conflict of interest.

## Publisher's note

All claims expressed in this article are solely those of the authors and do not necessarily represent those of their affiliated organizations, or those of the publisher, the editors and the reviewers. Any product that may be evaluated in this article, or claim that may be made by its manufacturer, is not guaranteed or endorsed by the publisher.
